# Gender stereotypes in leadership: Analyzing the content and evaluation of stereotypes about typical, male, and female leaders

**DOI:** 10.3389/fpsyg.2023.1034258

**Published:** 2023-01-27

**Authors:** Manuela Tremmel, Ingrid Wahl

**Affiliations:** ^1^Business Administration and Psychology, FernFH Distance-Learning University of Applied Sciences, Wiener Neustadt, Austria; ^2^Department of Communication, University of Vienna, Vienna, Austria

**Keywords:** leadership, agentic, communal, social representations, gender stereotype, associations, implicit measures, explicit measures

## Abstract

**Introduction:**

Previous research often examined gender stereotypes in leadership with ratings on predetermined gendered characteristics concerning leaders’ agency and communality (i.e., explicit measures). The aim of the present study was to broaden the understanding of gender stereotypes in leadership by taking more subtle approaches, that focus on what men and women actually ascribe to typical, male, and female leaders and how they implicitly evaluate them.

**Methods:**

An online survey collected (a) free associations which reflect social representations (e.g., dominant, empathic), (b) evaluations of the given associations as negative, neutral, or positive, and (c) ratings on Peabody’s semantic differential combining non-gendered adjective pairs to an evaluative component of a typical leader, a male leader, and a female leader.

**Results:**

Using the approach of social representations by analyzing 2,842 free associations from 194 participants shows the predominant gender stereotypes. Ratings of the free associations revealed that women evaluate characteristics associated with female leaders more negatively than those associated with typical leaders and male leaders. By contrast, using the evaluative component of non-gendered adjective pairs shows that typical and female leaders were often rated more positively than male leaders and that women were more likely to devalue male leaders.

**Discussion:**

Directly asking about leaders (i.e., associations) might retrieve participants’ gender stereotypes, whereas when using non-direct questions (i.e., evaluation component of adjective pairs) gender stereotypes might be less prominent. Thus, when evaluating leaders, practitioners and researchers should consider whether these evaluations were obtained explicitly or implicitly to assess potential influences of gender stereotypes.

## 1. Introduction

Globally, women held only 29% of senior management positions in 2020 (Thornton, 2020) and with increasing hierarchical positions in organizations women’s representation decreases (Mercer, 2020). Over time and across different countries, several studies showed that characteristics of successful leaders resemble stereotypical masculine characteristics, but not feminine characteristics, explaining the difficulties of women in reaching leadership positions (Schein, 1973, 2001; Brenner et al., 1989; Deal and Stevenson, 1998; Gmür, 2004; Castaño et al., 2019). Leaders are mainly seen to have masculine traits and characteristics similar to men and not to women (Koenig et al., 2011). Thus, it is easier for men to move up companies’ hierarchies to leadership positions (Eagly and Karau, 1991; Badura et al., 2018), whereas women face a glass ceiling that is hard to break through (Cotter et al., 2001).

The preference for male leaders over female leaders manifests itself in different occupational situations. For example, in hiring processes, leadership potential is overlooked when ranking female applicants (Player et al., 2019); in the absence of leadership experience, men prefer male applicants (Bosak and Sczesny, 2011), and men with low power rate female applicants worse and suggest a lower income (Hoover et al., 2019). Furthermore, in performance evaluations, female leaders are ranked as having as many positive attributes as male leaders; however, they also are presumed to have more negative attributes than male leaders, the attributes being mainly feminine (Smith et al., 2019). Promotions are given to women only when they have better performance ratings than men and standards for promotions are held more strictly for women than for men (Lyness and Heilman, 2006).

These inequalities regarding evaluations are frequently seen as the result of gender stereotypes among decision makers. As gender stereotypes may not always be conscious and evaluators may attempt to mask their gender stereotypes, it is difficult to scrutinize gender stereotypes with ratings on gendered characteristics; however, these explicit measures have been widely used in previous research. Applying methodological approaches that capture what people actually think about typical (i.e., leaders in general, without providing information on gender), male, and female leaders and obtaining implicit evaluations could overcome these limitations and provide new insights into gender stereotypes in leadership. Accordingly, this paper examines the content of gender stereotypes with social representations collected through free associations (i.e., explicit measure), and identifies evaluations of leaders by ratings of the free associations (i.e., explicit measure) and by combining ratings of non-gendered adjective pairs into an evaluative component (i.e., implicit measure). In addition, men are often the gatekeepers to leadership positions, restricting women’s access to upper-level positions. Therefore, gender differences in gender stereotypes concerning male and female leaders are crucial. Accordingly, this paper also examines gender differences in the content and evaluations obtained.

### 1.1. Men and women in leadership

Stereotypically, men are ascribed agentic characteristics describing them as aggressive, ambitious, dominant, forceful, independent, self-sufficient, self-confident, and prone to act as a leader. Stereotypes regarding women lie in communal characteristics portraying them as affectionate, helpful, kind, sympathetic, interpersonally sensitive, nurturant, and gentle (Koburtay et al., 2019). In general, communal characteristics are attributed to women equally by both genders; however, women are rated less agentic by men than by women (Hentschel et al., 2019). Role congruity theory suggests that agentic characteristics are congruent with characteristics of successful leaders, whereas communal characteristics are incongruent with characteristics of successful leaders. Accordingly, men are assumed to be more eligible than women for leadership positions (Eagly and Karau, 2002).

The misfit of stereotypical feminine characteristics to leadership characteristics results in negative behavior toward female leaders (Rudman et al., 2012). If female leaders show agentic characteristics, they are rated as less likable (Williams and Tiedens, 2016; Eichenauer et al., 2022), less hirable (Williams and Tiedens, 2016), and face more prejudice (Ferguson, 2018) than male leaders displaying agentic characteristics. The perception of dominant male leaders as the norm helps male leaders to be perceived as leaders; however, dominant female leaders are seen to be abnormal, which hinders them from being perceived as leaders (Kim et al., 2020). Female leaders in male-dominated working domains were especially devalued compared to male leaders (Eagly et al., 1992; Koch et al., 2015) and were found to be less competent, less influential, and less likely to have played a leadership role than their male counterparts (Heilman and Haynes, 2005). However, not only the misfit of characteristics but also failing to show stereotypical characteristics results in negative evaluations (Johnson et al., 2008).

Creating a fit of ascribed characteristics and characteristics of typical leaders influences leaders’ evaluations. Accordingly, male leaders are rated as more effective when the leadership role is defined in masculine terms and female leaders are rated as more effective when the leadership role is defined in feminine terms (Eagly et al., 1995). Providing information on the communal characteristics of successful female leaders in male-dominated working domains increased their ratings of likability, friendliness, and the desire to have them as leaders (Heilman and Okimoto, 2007). Female leaders who showed anger, because they witnessed harm done to another person (i.e., showing the communal characteristic of empathy), were evaluated as more effective and having more agentic and communal characteristics than corresponding male leaders (Keck, 2019). However, women applying for middle-management positions who stress their professional competence are seen to have better social competence than men stressing their professional competence (Steffens and Mehl, 2003). Also, successful female leaders at the top hierarchical level were described as more agentic and more communal than successful male leaders, suggesting additional stereotypes for top-level female leaders (Rosette and Tost, 2010). This requirement of agentic and communal characteristics produces a double-bind situation for female leaders, as they contradict each other (e.g., demanding and caring, authoritative and participative; Zheng et al., 2018).

Furthermore, men and women have different preferences regarding the sex of their leaders. Men devalue female leaders more than women do (Eagly et al., 1992; Deal and Stevenson, 1998; Cundiff and Komarraju, 2008) and have more prejudices against female leaders than women (Hoffmann and Musch, 2019). Accordingly, men think themselves to be more effective than women (Paustian-Underdahl et al., 2014). On the contrary, women show reduced favoritism for masculine characteristics in leaders (Brenner et al., 1989; Embry et al., 2008; Paris et al., 2009; Stoker et al., 2012). Thus, women do not engage in the masculine stereotyping of leadership, but instead see men and women similarly suitable for leadership positions (Schein, 2001; Boyce and Herd, 2003; Duehr and Bono, 2006; Berkery et al., 2013).

Previous experiences with female leaders have positive effects on female leaders’ perception. People without work experience hold more masculine stereotypes about leaders than people with work experience (Koenig et al., 2011) and people experienced in working with male and female leaders saw a greater accordance between women and leaders (Berkery et al., 2013). Experienced professionals are less biased toward male applicants (Koch et al., 2015). In laboratory experiments and assessment studies, stereotypical gender ascriptions were found, suggesting that male leaders are task-oriented (i.e., agentic) and female leaders are person-oriented (i.e., communal). However, these findings were not found in studies conducted in organizational contexts (Eagly and Johnson, 1990). Individuals with positive experiences with female leaders found women to have better leadership skills than people with no prior positive experiences with female leaders (Duehr and Bono, 2006). Furthermore, people with female leaders and people working in companies with many female leaders show a reduced favoritism for masculine characteristics in leaders (Stoker et al., 2012). Additionally, women who came into contact with female leaders show reduced automatic stereotypical assumptions about female leaders (Dasgupta and Asgari, 2004).

There seem to be changes in leadership stereotypes over time; however, they are rather small (Eagly et al., 2020). Studies analyzing obituaries over a period of several decades found a change in stereotypes ascribed to male and female leaders, indicating that stereotypes of male leaders come closer to stereotypes of female leaders and stereotypes of female leaders come closer to stereotypes of male leaders (Rodler et al., 2001; Hartl et al., 2013). Leaders are nowadays seen to be more androgynous (Koenig et al., 2011; Kark et al., 2012) and additionally hold stereotypical feminine characteristics (e.g., individualized consideration in transformational leadership suggesting that leaders should be empathic with employees’ needs; Sczesny et al., 2004; Eagly and Sczesny, 2009; Vinkenburg et al., 2011). Moreover, women are seen to be more androgynous holding stereotypical masculine and feminine characteristics (Duehr and Bono, 2006; Berkery et al., 2013). Accordingly, stereotypical male and stereotypical feminine characteristics in leadership were desired by an Australian corporate sample and female leaders were ascribed characteristics that are stereotypically associated with men (Griffiths et al., 2019). However, stereotypical masculine characteristics are still seen as key prerequisites for successful leadership and leaders’ stereotypical feminine characteristics are rather seen as nice-to-have add-ons (Vial and Napier, 2018).

### 1.2. Measuring gender stereotypes

Depending on whether gender stereotypes are measured explicitly or implicitly, different information is assessed, which might result in differing assessments. Traditionally, explicit measures have been used, which are mostly self-report ratings on items generated by researchers that cannot capture stereotypes specific to individual participants (Kite et al., 2008). These ratings refer, for example, to how likely either a person (i.e., person in general, without providing information on gender), a man, or a woman exhibits gendered characteristics regarding traits, role behaviors, occupations, and physical attributes (e.g., Haines et al., 2016), or to estimating the percent prevalence of stereotypical characteristics (McCauley and Stitt, 1978). In order to capture stereotypes of individual participants, Eagly and Mladinic (1989) asked their participants to indicate typical characteristics of men and women in free responses (i.e., free associations), which were then rated by the participants themselves. Subsequently, only the ratings obtained were analyzed, not the content of these responses; however, analyzing the given verbal responses could shed light on prevailing stereotypes. Drawing on Mladinic’s approach, the present study also uses free associations to explicitly gauge the content and the evaluation of gender stereotypes.

Implicit measures, on the other hand, influence participants less as they indirectly ask about participants’ assessments (Kite et al., 2008). Implicit measures of gender stereotypes range from (a) speed of response in evaluating masculine and feminine characteristics for men and women, to (b) evaluating books where the author is either male or female, to (c) judging whether a name is remembered as famous, to (d) judging which stereotypical characteristic would alter judgements about a male or female person (for an overview see Greenwald and Banaji, 1995). For these measures, however, specific measurement tools or manipulations are needed, which complicates their use for specific research questions and in questionnaire studies. Another implicit measure for capturing evaluations of different groups is Peabody’s semantic differential. Although ratings are used, the evaluations obtained are implicit as participants are unaware of the evaluative component; however, evaluations are more easily obtained than evaluations of other implicit measures. Thus, the present study uses Peabody’s semantic differential to examine evaluations of leaders implicitly.

### 1.3. Social representations

Individuals construct their reality through communication in everyday life (e.g., discussions, debates, and media reports). This results in social representations defined as a set of concepts, statements, and explanations about the concerned social object from a layperson’s perspective. For example, when people ignorant of the term gender follow a discussion about gender they form beliefs of the term’s meaning (e.g., sex, socially constructed) on the basis of this communication. Now, these people can enter discussions with other people, who have also previously formed beliefs about the meaning of gender. As they can assume that they have the same – socially shared – beliefs about gender (i.e., social representations) they can start their discussion without having to define the meaning of gender first, as they have already an agreement on the term’s meaning. As an outcome of a group process, social representations are influenced by peers, past experiences, and social backgrounds. This leads to different social representations depending on an individual’s group membership (e.g., people reading feminist literature might have other social representations about gender than people who do not consume such literature). Hence, social representations indicate how different groups think about and which common perception they have about specific phenomena (i.e., individuals, events, objects, etc.; Moscovici, 1981, 1984, 2001).

Social representations consist of a central core and peripheral elements. The central core is the homogeneous, stable, and coherent collectively shared basis of a group’s social representation. For example, the central core of the term gender would be “social construct,” “man,” and “woman.” Peripheral elements integrate individual experiences, are sensitive to immediate contexts, and are therefore more flexible and can be inconsistent (Abric, 1993). For example, a person’s peripheral elements of gender could be “cis” and “non-cis” as they just read an article on the subject. Spontaneous free associations to stimulus words (e.g., financial crisis, taxes, non-profit organization, euro) provided have been used to examine social representations in varying contexts (e.g., social representations of financial crisis: unemployment, bank, credit; social representations of non-profit organization: help, donation, social; *cf.* Kirchler, 1998; Meier and Kirchler, 1998; Wagner et al., 1999; Gangl et al., 2012; Rodrigues et al., 2015; Marx et al., 2019). Associations that represent the central core of a stimulus are mentioned frequently and early in the chain of associations, whereas associations mentioned less frequently and/or later represent peripheral elements (Vergès and Bastounis, 2001). Thus, the approach of free associations provides insights into unstructured and latent views unaffected by socially desired behavior (Kulich et al., 2005).

Previous studies examining masculine and feminine characteristics used mainly explicit measures with self-ratings on items created by researchers, which cannot capture stereotypes held by individuals. However, assumptions anchored in society are difficult to study and are influenced easily by these methods (Wagner et al., 1999). To receive less influenced lay perceptions and, thus, to broaden the insight into prevailing gender stereotypes the present study uses the approach of social representations collected through free associations. In addition, ratings on the given free associations provide information about the evaluation of gender stereotypes. As belonging to different social groups can influence social representations, gender differences are also examined.

### 1.4. Peabody’s semantic differential

Peabody (1985) developed a measure assessing whether people are evaluated positively or negatively without asking participants directly about their evaluations. To obtain this evaluative component, ratings of two bi-polar adjective pairs describing the same characteristic with opposing evaluations are combined. For example, the first adjective pair is extravagant vs. thrifty and the matching second pair is stingy vs. generous. Thrifty as well as stingy describe a person who spends little money, whereas extravagant and generous describe a person who spends a lot of money. Although, thrifty and generous describe opposites, both are evaluated positively, while extravagant and stingy also describe opposites but are evaluated negatively. Thus, these ratings have an underlying evaluative component which can be determined by combining the ratings of the two matching adjective pairs.

Collecting implicit evaluations might lead to evaluations less influenced by gender stereotypes than explicit evaluations. To this end, the semantic differential of Peabody (1985) with its evaluative component is used in the present paper. Since men and women might differ in their implicit evaluation, gender differences are also examined.

### 1.5. The present study

The motivation of the present study was to capture gender stereotypes actually present in leadership and to obtain explicit and implicit evaluations regarding these stereotypes. To this end, the more subtle approach of social representations obtained with free associations and Peabody’s implicit evaluative component derived from ratings of non-gendered adjectives were used. It is expected that the assumptions of role congruity theory (Eagly and Karau, 2002) are still prevailing. This is to say that typical leaders and male leaders tend to be ascribed mainly agentic characteristics in the central core of social representations, while female leaders’ central core consists mainly of communal characteristics. However, stereotypes of male and female leaders converge over time and masculine as well as feminine characteristics are desirable for leaders (Rodler et al., 2001; Sczesny et al., 2004; Eagly and Sczesny, 2009; Koenig et al., 2011; Vinkenburg et al., 2011; Kark et al., 2012; Hartl et al., 2013; Griffiths et al., 2019). It is assumed that in male leaders’ central core also communal characteristics will be found, whereas the central core for female leaders also includes agentic characteristics. Women see female leaders as similarly suitable as male leaders for leadership positions (Schein, 2001; Boyce and Herd, 2003; Duehr and Bono, 2006; Berkery et al., 2013). Accordingly, it is expected that women’s central cores for typical and female leaders will be rather similar, whereas men’s central cores for typical leaders and female leaders are expected to differ.

In addition to analyzing the content of gender stereotypes ratings of free associations and non-gendered adjective pairs can be also used to scrutinize gender stereotypes. Ratings of free associations (i.e., neutrality indices, polarity indices) reveal the evaluation of the produced associations as either positive or negative and reflect in a further step the evaluation of the used stimulus. By contrast, the implicitly obtained evaluative component of non-gendered adjective pairs (Peabody, 1985) indicates directly whether the given stimuli are rated as either positive or negative. Both approaches allow analyzing evaluations without providing participants with explicitly gendered material. Previous research found that men frequently devalue female leaders (Eagly et al., 1992; Deal and Stevenson, 1998; Hoffmann and Musch, 2019). Accordingly, it is expected that ratings of free associations and evaluations of non-gendered adjective pairs will reveal that men evaluate female leaders worse than both male and typical leaders compared to women.

## 2. Methods

### 2.1. Material and procedure

An introduction letter which ensured participants’ anonymity and included a link to the online questionnaire, was distributed in the first author’s professional and private networks. Participants could cancel their participation at any time and were not remunerated. Only respondents who indicated at the beginning of the online questionnaire that they had work experience were included.

An online questionnaire was developed which proceeded in two phases. In the first phase, participants were asked on separate pages to think about a typical, a male, or a female leader. Stimuli were presented in a system-generated random order. On each page, they were instructed to associate freely and write down the idea/s that came to their mind for the stimulus presented. For each stimulus, a maximum of 10 associations could be specified. After reporting free associations to a stimulus, participants were asked to indicate whether each association was positive, neutral, or negative on a three-point scale.

In the second phase, Peabody’s semantic differential was used. Participants were presented with the list of all 32 adjective pairs from Peabody (1985) on separate pages for each stimulus (i.e., typical, male, and female leader) in the same order as in the first phase. The adjectives of each pair represented the end poles of the bi-polar rating scale and participants rated on a seven-point answering format ranging from −3 for the less favorable adjective to +3 for the more favorable adjective. For all stimuli the order of the adjective pairs and the position of the adjectives were the same. Additionally, data on the participants’ gender, age, leadership position, and educational level were collected. The mean response time was 12.9 min (*SD* = 4.0, *Mdn* = 12.4; *25% quantile* = 10.2, *75% quantile* = 15.4).

### 2.2. Participants

The online questionnaire was started by 549 participants and completed by 194 participants (67.0% female and 30.4% male; 2.6% did not indicate their gender), whose ages ranged from 21 to 65 years (*M* = 44.5, *SD* = 10.3, *Mdn* = 46.0). The majority of participants (74.2%) worked full time (i.e., at least 37.5 h per week), 23.7% worked part-time (i.e., less than 37.5 h per week), and 2.1% worked irregularly or did not indicate their weekly working hours. Most participants (72.7%) did not hold a leadership position, 26.3% held a leadership position, and 1.0% did not respond to this question. A college degree was held by 29.9% of the participants, 50.0% had graduated from high school, 17.5% indicated another level of education, and 2.6% did not indicate their level of education. Participants’ demographics were collected at the end of the questionnaire; therefore, dropouts could not be analyzed with respect to these variables.

*A posteriori* power analyses for analyses of variance with one within-subjects factor with three measuring points and one between-subjects factor with two groups was conducted. With a sample size of n = 194 and a significance level of 0.05 the power (1-β) for small effect sizes (0.10) is 0.22, for medium effect sizes (0.25) it is 0.88, and for high effect sizes (0.40) it is 1.00. For the smallest significance level corrected for Bonferroni-Holm (i.e., 0.05 / 17 = 0.0029) the power for small effects (0.10) is 0.03, for medium effects (0.25) it is 0.56, and for high effect sizes (0.40) it is 0.99 (c.f. Faul et al., 2009).

### 2.3. Data analyses of associations and adjective pairs

The associations produced spontaneously were rated by the participants as positive, neutral, or negative. The numbers of positive, neutral, and negative evaluations can be combined to a polarity index, indicating how positive or negative associations to a stimulus were rated. The polarity index is calculated by the number of positive associations minus the number of negative associations in relation to the total number of associations and ranges from −1 for negative evaluations to +1 for positive evaluations (formulas: number of total associations = number of positive associations + number of negative associations + number of neutral associations; polarity index = (number of positive associations − number of negative associations) / number of total associations; de Rosa, 1995, as cited in Kirchler, 2011).

Associations rated as either positive, neutral, and negative can also be combined to a neutrality index. This index indicates whether the stimulus has strongly polarized or neutral associations. It is calculated by the number of neutral associations minus the number of positive and negative associations in relation to the total number of associations and ranges from −1 for strongly polarized associations to +1 for predominantly neutral associations {formulas: total number of associations = number of positive associations + number of negative associations + number of neutral associations; neutrality index = [number of neutral associations – (number of positive associations + number of negative associations)] / total number of associations; de Rosa, 1995, as cited in Kirchler, 2011}.

To obtain the evaluative component of adjective pairs, the ratings of two connected pairs were combined to an evaluation score according to Peabody’s instructions [i.e., recoding each pair so that −3 indicates the negative adjective and + 3 indicates the positive adjective; building the sum of the two connected pairs and dividing the sum by two; formula: evaluation score of a pair = (evaluation of first pair + evaluation of second pair) / 2]. For example, a participant’s rating on the adjective pair extravagant – thrifty is +2 and the same participant’s rating on the adjective pair stingy – generous is +1. For both adjective pairs the positively evaluated adjective is on the right-hand side; therefore, no recoding is needed (i.e., recoding is needed if the positively evaluated adjective of an adjective pair is on the left-hand side; however, in the current study no recoding was necessary). Following the formula, the evaluation score of the two pairs is (2 + 1) / 2 = +1.50 which indicates that the underlying evaluation is positive as it is above 0; values below 0 indicate negative evaluations.

Evaluation scores of the adjective pairs were scrutinized with 17 mixed analyses of variance. Each analysis included an independent within-subjects factor stimulus consisting of the respective evaluation scores of typical, male, and female leaders and participants’ gender as independent between-subjects factor. Evaluation scores were formed with the pairs extravagant–thrifty / stingy–generous, impulsive–self-controlled / inhibited–spontaneous, frivolous–serious / grim–jolly, gullible–skeptical / distrustful–trusting, lax–firm / severe–lenient, vacillating–persistent / inflexible–flexible, undiscriminating–selective / choosy–broad-minded, rash–cautious / timid–bold, agitated–calm / inactive–active, aggressive–peaceful / passive–forceful, conceited–modest / unassured–self-confident, uncooperative–cooperative / conforming–independent, tactless–tactful / devious–frank, impractical–practical / opportunistic–idealistic, and deplorable–admirable / not likable–likable.

To compare the polarity indices, a mixed analysis of variance was conducted, with the independent within-subjects factor stimulus including the polarity indices of typical, male, and female leaders and participants’ gender as the independent between-subjects factor (i.e., one within-subjects factor with three measuring points and one between-subjects factor; *cf.* Table 1). Further, to examine the neutrality indices, a mixed analysis of variance was conducted, with the independent within-subjects factor stimulus consisting of neutrality indices of typical, male, and female leaders and participants’ gender as the independent between-subjects factor. Peabody (1985) also used the adjective pairs lazy–hard working and stupid–intelligent which he did not combine to evaluations scores. The last two mixed analyses of variance compared data of these adjective pairs. The independent within-subjects factor was called stimulus and consisted of the adjective pairs lazy–hard working and stupid–intelligent of typical, male, and female leaders, respectively. Participants’ gender represents the independent between-subjects factor.

**Table 1:** Estimated marginal means and standard errors of polarity indices and neutrality indices for the stimuli “typical leader,” “male leader,” and “female leader” by participants’ gender.

Stimulus	Typical leader		Male leader		Female leader	
Index	Men (*n* = 58)	Women (*n* = 130)	Men (*n* = 58)	Women (*n* = 130)	Men (*n* = 58)	Women (*n* = 130)
Polarity index	0.51 (0.07)	0.35 (0.05)	0.28 (0.07)	0.40 (0.05)	0.34 (0.08)	0.09 (0.05)
Neutrality index	−0.53 (0.07)	−0.64 (0.05)	−0.56 (0.06)	−0.70 (0.04)	−0.52 (0.08)	−0.49 (0.05)

Values outside parentheses are estimated marginal means ranging from −1 (= negative evaluation for polarity index and non-neutral evaluation for neutrality index) to + 1 (= positive evaluation for polarity index and neutral evaluation for neutrality index); values in parentheses indicate standard errors.

As 17 analyses were conducted to examine evaluation scores (*cf.* Table 2), *Alpha* was adjusted according to the Bonferroni–Holm method (Hemmerich, 2016). For all mixed analyses of variance, the main effects of “stimulus” and “participants’ gender” as well as the interaction effect of “stimulus x participants’ gender” was examined. For significant main effects of “stimulus” and for significant interaction effects pairwise comparisons with Bonferroni *post hoc* tests were conducted.

**Table 2:** Estimated marginal means and standard errors of the evaluation scores for the stimuli “typical leader,” “male leader,” and “female leader” by participants’ gender.

Stimulus	*n*		Typical leader		Male leader		Female leader	
Paired items’ underlying evaluation scores	Men	Women	Men	Women	Men	Women	Men	Women
Extravagant–thrifty / stingy–generous	57	126	0.28 (0.11)	0.36 (0.07)	−0.02 (0.12)	0.00 (0.08)	0.33 (0.13)	0.73 (0.09)
Impulsive–self-controlled/ inhibited–spontaneous	57	126	1.01 (0.15)	1.04 (0.10)	0.68 (0.15)	0.45 (0.10)	0.33 (0.15)	0.59 (0.10)
Frivolous–serious / grim–jolly	57	126	0.75 (0.12)	0.67 (0.08)	0.37 (0.13)	0.29 (0.08)	0.55 (0.13)	0.76 (0.09)
Gullible–skeptical / distrustful–trusting	57	126	0.68 (0.11)	0.83 (0.08)	0.64 (0.11)	0.55 (0.08)	0.46 (0.12)	0.71 (0.08)
Lax–firm / severe–lenient	57	126	0.78 (0.11)	0.80 (0.08)	0.68 (0.12)	0.43 (0.08)	0.56 (0.13)	0.94 (0.09)
Vacillating–persistent / inflexible–flexible	57	126	0.87 (0.16)	0.86 (0.11)	0.61 (0.16)	0.32 (0.11)	0.34 (0.16)	0.94 (0.10)
Undiscriminating–selective / choosy–broad-minded	57	126	0.90 (0.14)	1.02 (0.10)	0.84 (0.13)	0.58 (0.09)	0.79 (0.14)	1.22 (0.10)
Rash–cautious / timid–bold	57	126	0.91 (0.12)	1.01 (0.08)	0.69 (0.12)	0.77 (0.08)	0.74 (0.12)	0.81 (0.08)
Agitated–calm / inactive–active	57	126	0.77 (0.14)	0.77 (0.09)	0.74 (0.13)	0.48 (0.08)	0.35 (0.13)	0.65 (0.09)
Aggressive–peaceful / passive–forceful	55	124	0.84 (0.12)	0.84 (0.08)	0.60 (0.13)	0.39 (0.08)	0.62 (0.13)	1.06 (0.09)
Conceited–modest / unassured–self-confident	57	125	0.79 (0.13)	0.82 (0.09)	0.59 (0.13)	0.56 (0.09)	0.37 (0.15)	0.56 (0.10)
Uncooperative–cooperative / conforming–independent	55	126	0.86 (0.17)	0.93 (0.11)	0.57 (0.18)	0.38 (0.12)	0.66 (0.16)	0.92 (0.11)
Tactless–tactful / devious–frank	57	125	0.99 (0.19)	0.87 (0.13)	0.61 (0.17)	0.08 (0.12)	0.86 (0.18)	1.08 (0.12)
Impractical–practical / opportunistic–idealistic	56	124	0.96 (0.15)	0.82 (0.10)	0.70 (0.15)	0.48 (0.10)	0.35 (0.16)	0.88 (0.11)
Deplorable–admirable / not likable–likable	57	126	0.77 (0.17)	0.75 (0.11)	0.62 (0.17)	0.48 (0.11)	0.88 (0.17)	1.12 (0.12)
Lazy–hard-working	57	126	1.70 (0.17)	1.66 (0.11)	1.46 (0.17)	1.10 (0.12)	1.63 (0.16)	2.08 (0.11)
Stupid–intelligent	57	126	1.60 (0.17)	1.75 (0.11)	1.44 (0.17)	1.28 (0.11)	1.49 (0.16)	1.93 (0.11)

Adjectives on the left are negatively evaluated and adjectives on the right are positively evaluated; values outside parentheses are estimated marginal means ranging from −3 (= negative evaluation, left adjective of the pair) to + 3 (= positive evaluation, right adjective of the pair); values in parentheses indicate standard errors.

Mixed analyses of variance were conducted, although, assumptions of parametric tests were violated (*cf.* Table 3 for information on violations of assumptions). First, analyses of variance are commonly used and understood, whereas its non-parametric equivalent is mostly unknown. Second, analyses of variance allow easy access to interaction effects. Third, analyses of variance are robust to these violations when sample sizes are sufficient (*cf.* Bortz and Schuster, 2010). In case the assumption of sphericity was violated, the Greenhouse–Geisser test was used.

**Table 3:** Test statistics of tests on evaluations of associations and evaluation of adjective pairs.

Evaluations of associations						
	Violated assumptions for parametric testing	*F*	*df1*	*df2*	*p*	η^2^
*Evaluation of polarity indices*	all DVs NN, BM, EV for typical leader					
Within-subjects effects						
Stimulus		7.06	2.00	372.00	***	0.037
Stimulus x participants’ gender		5.22	2.00	372.00	0.006	0.027
Between-subjects effects						
Participants’ gender		2.97	1.00	186.00	0.087	0.016
*Evaluation of neutrality indices*	all DVs NN, BM, MA					
Within-subjects effects						
Stimulus		3.13	1,89	351.61	0.048	0.017
Stimulus x participants’ gender		1.54	1,89	351.61	0.217	0.008
Between-subjects effects						
Participants’ gender		1.53	1.00	186.00	0.218	0.008
Evaluation of adjective pairs						
*Evaluation of extravagant–thrifty / stingy–generous*	all DVs NN, MA					
Within-subjects effects						
Stimulus		16.00	1.79	323.11	***	0.081
Stimulus x participants’ gender		2.40	1.79	323.11	0.099	0.013
Between-subjects effects						
Participants’ gender		3.46	1.00	181.00	0.065	0.019
*Evaluation of impulsive–self-controlled / inhibited–spontaneous*	all DVs NN					
Within-subjects effects						
Stimulus		12.70	2.00	362.00	***	0.006
Stimulus x participants’ gender		2.16	2.00	362.00	0.117	0.012
Between-subjects effects						
Participants’ gender		0.03	1.00	181.00	0.869	0.000
*Evaluation of frivolous–serious / grim–jolly*	all DVs NN, MA					
Within-subjects effects						
Stimulus		9.61	1.71	308.66	***	0.050
Stimulus x participants’ gender		1.54	1.71	308.66	0.217	0.008
Between-subjects effects						
Participants’ gender		0.02	1.00	181.00	0.877	0.000
*Evaluation of gullible–skeptical / distrustful–trusting*	all DVs NN, MA					
Within-subjects effects						
Stimulus		2.63	1.91	346.22	0.074	0.014
Stimulus x participants’ gender		2.23	1.91	346.22	0.137	0.012
Between-subjects effects						
Participants’ gender		1.05	1.00	181.00	0.306	0.006
*Evaluation of lax–firm / severe–lenient*	all DVs NN, BM, MA					
Within-subjects effects						
Stimulus		3.36	1.91	345.88	0.038	0.018
Stimulus x participants’ gender		5.02	1.91	345.88	0.008	0.027
Between-subjects effects						
Participants’ gender		0.32	1.00	181.00	0.575	0.002
*Evaluation of vacillating–persistent / inflexible–flexible*	all DVs NN, BM, MA					
Within-subjects effects						
Stimulus		5.42	1.90	343.81	0.006	0.029
Stimulus x participants’ gender		6.81	1.93	343.81	0.002	0.036
Between-subjects effects						
Participants’ gender		0.62	1.00	181.00	0.431	0.003
*Evaluation of undiscriminating–selective / choosy–broad-minded*	all DVs NN					
Within-subjects effects						
Stimulus		4.49	2.00	362.00	0.012	0.024
Stimulus x participants’ gender		5.45	2.00	362.00	0.005	0.029
Between-subjects effects						
Participants’ gender		0.73	1.00	181.00	0.393	0.004
*Evaluation of rash–cautious / timid–bold*	all DVs NN, BM, MA					
Within-subjects effects						
Stimulus		4.40	1.77	321.06	0.016	0.024
Stimulus x participants’ gender		0.01	1.77	321.06	0.983	0.000
Between-subjects effects						
Participants’ gender		0.63	1.00	181.00	0.429	0.003
*Evaluation of agitated–calm / inactive–active*	all DVs NN, BM, MA					
Within-subjects effects						
Stimulus		3.83	1.84	333.61	0.026	0.021
Stimulus x participants’ gender		4.03	1.84	333.61	0.022	0.022
Between-subjects effects						
Participants’ gender		0.01	1.00	181.00	0.928	0.000
*Evaluation of aggressive–peaceful / passive–forceful*	all DVs NN, MA					
Within-subjects effects						
Stimulus		9.69	1.88	332.52	***	0.052
Stimulus x participants’ gender		6.83	1.88	332.52	0.002	0.037
Between-subjects effects						
Participants’ gender		0.55	1.00	177.00	0.460	0.003
*Evaluation of conceited–modest / unassured–self-confident*	all DVs NN, BM, MA					
Within-subjects effects						
Stimulus		4.91	1.81	324.82	0.010	0.027
Stimulus x participants’ gender		0.56	1.81	324.82	0.552	0.003
Between-subjects effects						
Participants’ gender		0.35	1.00	180.00	0.558	0.002
*Evaluation of uncooperative–cooperative / conforming–independent*	all DVs NN, MA					
Within-subjects effects						
Stimulus		5.44	1.92	344.31	0.005	0.030
Stimulus x participants’ gender		1.55	1.92	344.31	0.215	0.009
Between-subjects effects						
Participants’ gender		0.13	1.00	179.00	0.717	0.001
*Evaluation of tactless–tactful / devious–frank*	all DVs NN					
Within-subjects effects						
Stimulus		13.98	2.00	360.00	***	0.072
Stimulus x participants’ gender		3.97	2.00	360.00	0.020	0.022
Between-subjects effects						
Participants’ gender		0.90	1.00	180.00	0.345	0.005
*Evaluation of impractical–practical / opportunistic–idealistic*	all DVs NN, BM, MA					
Within-subjects effects						
Stimulus		4.57	1.77	315.43	0.014	0.025
Stimulus x participants’ gender		6.89	1.77	315.43	0.002	0.037
Between-subjects effects						
Participants’ gender		0.19	1.00	178.00	0.661	0.001
*Evaluation of deplorable–admirable / not likable–likable*	all DVs NN, MA					
Within-subjects effects						
Stimulus		6.85	1.79	323.12	0.002	0.036
Stimulus x participants’ gender		1.33	1.79	323.12	0.264	0.007
Between-subjects effects						
Participants’ gender		0.03	1.00	181.00	0.870	0.000
*Evaluation of lazy–hard-working*	all DVs NN					
Within-subjects effects						
Stimulus		14.66	2.00	362.00	***	0.075
Stimulus x participants’ gender		6.86	2.00	362.00	0.001	0.037
Between-subjects effects						
Participants’ gender		0.01	1.00	181.00	0.913	0.000
*Evaluation of stupid–intelligent*	all DVs NN					
Within-subjects effects						
Stimulus		7.21	2.00	362.00	***	0.038
Stimulus x participants’ gender		4.30	2.00	362.00	0.014	0.023
Between-subjects effects						
Participants’ gender		0.79	1.00	181.00	0.375	0.004

***p < 0.001; NN indicates that the dependent variable is not normally distributed (i.e., significant Shapiro–Wilk test); BM indicates that covariances were not equal (i.e., significant Box-M test); MA indicates that sphericity was not assumed (i.e., significant Mauchly test of sphericity) and the measure of Greenhouse–Geisser was used; EV indicates that the error variance of the dependent variable is not equal; DV indicates dependent variable.

## 3. Results

### 3.1. Content of free associations

In total, participants indicated 2,842 free associations (typical leader: 1031, male leader: 880, female leader: 931). After correcting for typos and aligning the synonyms (i.e., changing associations with the same meaning to the same association, for example, the association “just” was changed to “fair”), the total number of different associations was 847 (typical leader: 429, male leader: 416, female leader: 403). As the same associations were partly given for two or more stimuli, the total number of associations does not reflect the sum of associations given to each stimulus. Associations were then translated from German to English by the authors.

**Figure 1:**
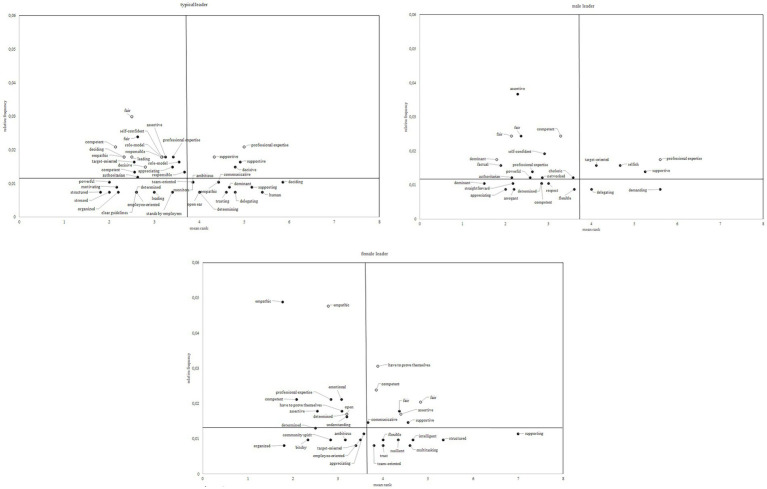
Relative frequencies and mean ranks of associations to the stimuli “typical leader,” “male leader,” and “female leader” by participants’ gender. *Note:* Only associations with frequencies above 4 were included.

First, frequencies for each association were calculated. Second, for each association it was regarded how many participants associated it as their first, their second and so on association (i.e., production process). Third, for each association mean ranks of the production process were calculated indicating at which point the association was made on average (formula: mean rank = (frequency of association on the first place * 1 + frequency of association on the second place * 2 + frequency of association on the third place * 3 + … + frequency of the association on the tenth place * 10) / total frequency of association). Figure 1 compares the associations with typical, male, and female leaders as a function of participants’ gender by plotting associations’ frequencies and mean ranks in the production process. Plots were divided by the authors after carefully considering frequencies, mean ranks, and resulting number of associations in the central core. Thresholds were implemented horizontally for frequencies below and above 1% and vertically for mean ranks below and above 3.7, resulting in four sections. Associations building the central core of social representations are found in the upper left section of each plot, whereas the other sections represent peripheral elements. The comparison of the central core regarding typical leaders shows that both men and women associate “fair,” “competent,” “role-model,” and “responsible” with typical leaders. Men describe typical leaders additionally as “empathic,” “deciding,” and “leading.” Women’s core social representations to typical leaders are “target-oriented,” “self-confident,” “assertive,” “appreciating,” “professional expertise,” and “authoritarian.”

Social representations forming the central core of a male leader was “fair” for both women and men. Men also have the core representations “competent” and “dominant” with male leaders, and women’s central core consists of the characteristics “assertive,” “self-confident,” “factual,” “powerful,” “networked,” “short-tempered,” and “professional expertise” with male leaders.

Core social representations for female leaders are “empathic” and “determined” by both women and men. Men’s only additional core social representation with female leaders was “open,” and women’s core included “competent,” “emotional,” “professional expertise,” “have to prove themselves,” “assertive,” and “understanding” with female leaders.

### 3.2. Evaluation of free associations

Tests on the polarity indices indicated a significant interaction effect, [*F*(2,185) = 5.08, *p* = 0.007, η^2^ = 0.052]. The results showed that women evaluate their associations for female leaders more negatively than men evaluate their associations for female leaders. Further, men evaluate their associations for typical leaders more positively than for male leaders; however, no difference between evaluations of male leaders and female leaders and between typical leaders and female leaders were revealed. Women’s associations for female leaders were more negative than associations for typical leaders as well as male leaders; however, women evaluated typical and male leaders equally.

Tests on the neutrality indices indicated a significant main effect of the within-subjects factor comparing the neutrality indices of typical, male, and female leaders, *F*(2,185) = 3.87, *p* = 0.023, η^2^ = 0.040. The results showed that evaluations of typical leaders do not differ from male and female leaders; however, female leaders are evaluated more neutrally than male leaders. Table 1 shows the estimated marginal means and standard errors of polarity indices and neutrality indices for the factor stimulus split according to participants’ gender. Information on test statistics on polarity and neutrality indices are provided in Table 3. Values of mean differences and *p* values of all pairwise comparisons are depicted in Table 4.

**Table 4:** Mean differences and *p*-values of pairwise comparisons of indices and adjective pairs’ significant main effects of the independent within-subjects factor stimulus and significant interaction effects of the within-subjects factor stimulus and the between-subjects factor participants’ gender.

Index or adjective pair	Subgroup	Comparisons	Mean difference	*p*
Main effects of stimulus				
Neutrality index		Typical leader vs. male leader	0.05	0.359
		Typical leader vs. female leader	−0.08	0.160
		Male leader vs. female leader	−0.13	0.006
Extravagant–thrifty / stingy–generous		Typical leader vs. male leader	0.33	***
		Typical leader vs. female leader	−0.21	0.026
		Male leader vs. female leader	−0.54	***
Impulsive–self-controlled / inhibited–spontaneous		Typical leader vs. male leader	0.45	***
		Typical leader vs. female leader	0.57	***
		Male leader vs. female leader	0.11	0.384
Frivolous–serious / grim–jolly		Typical leader vs. male leader	0.38	***
		Typical leader vs. female leader	0.05	0.600
		Male leader vs. female leader	−0.33	0.003
Rash–cautious / timid–bold		Typical leader vs. male leader	0.23	***
		Typical leader vs. female leader	0.19	0.028
		Male leader vs. female leader	−0.05	0.625
Conceited–modest / unassured–self-confident		Typical leader vs. male leader	0.23	0.015
		Typical leader vs. female leader	0.34	0.002
		Male leader vs. female leader	0.11	0.390
Uncooperative–cooperative / conforming–independent		Typical leader vs. male leader	0.42	0.42
		Typical leader vs. female leader	0.11	0.407
		Male leader vs. female leader	−0.31	0.032
Tactless–tactful / devious–frank		Typical leader vs. male leader	0.59	***
		Typical leader vs. female leader	−0.40	0.764
		Male leader vs. female leader	−0.63	***
Deplorable–admirable / not likable–likable		Typical leader vs. male leader	0.21	0.036
		Typical leader vs. female leader	−0.24	0.060
		Male leader vs. female leader	−0.45	0.001
Stupid-intelligent		Typical leader vs. male leader	0.32	0.001
		Typical leader vs. female leader	−0.04	0.733
		Male leader vs. female leader	−0.35	0.001
Interaction effects of stimulus and participants’ gender				
Polarity index	Women	Typical leader vs. male leader	−0.04	1.000
		Typical leader vs. female leader	0.27	***
		Male leader vs. female leader	0.31	***
	Men	Typical leader vs. male leader	0.23	0.073
		Typical leader vs. female leader	0.17	0.225
		Male leader vs. female leader	−0.06	1.000
	Typical leader	Women vs. men	−0.16	0.068
	Male leader	Women vs. men	0.11	0.198
	Female leader	Women vs. men	−0.25	0.007
Vacillating–persistent / inflexible–flexible	Women	Typical leader vs. male leader	0.54	***
		Typical leader vs. female leader	−0.08	1.000
		Male leader vs. female leader	−0.62	***
	Men	Typical leader vs. male leader	0.26	0.497
		Typical leader vs. female leader	0.53	0.022
		Male leader vs. female leader	0.26	0.736
	Typical leader	Women vs. men	−0.01	0.970
	Male leader	Women vs. men	−0.29	0.130
	Female leader	Women vs. men	0.60	0.002
Undiscriminating–selective / choosy–broad-minded	Women	Typical leader vs. male leader	0.44	***
		Typical leader vs. female leader	−0.20	0.309
		Male leader vs. female leader	−0.64	***
	Men	Typical leader vs. male leader	0.05	1.000
		Typical leader vs. female leader	0.11	1.000
		Male leader vs. female leader	0.05	1.000
	Typical leader	Women vs. men	0.13	0.465
	Male leader	Women vs. men	−0.26	0.105
	Female leader	Women vs. men	0.43	0.012
Aggressive–peaceful / passive–forceful	Women	Typical leader vs. male leader	0.45	***
		Typical leader vs. female leader	−0.22	0.104
		Male leader vs. female leader	−0.67	***
	Men	Typical leader vs. male leader	0.24	0.209
		Typical leader vs. female leader	0.22	0.494
		Male leader vs. female leader	−0.02	1.000
	Typical leader	Women vs. men	0,00	0.987
	Male leader	Women vs. men	−0.21	0.167
	Female leader	Women vs. men	0.44	0.004
Impractical–practical / opportunistic–idealistic	Women	Typical leader vs. male leader	0.34	0.003
		Typical leader vs. female leader	−0.06	1.000
		Male leader vs. female leader	−0.40	0.016
	Men	Typical leader vs. male leader	0.26	0.283
		Typical leader vs. female leader	0.61	0.003
		Male leader vs. female leader	0.35	0.301
	Typical leader	Women vs. men	−0.13	0.475
	Male leader	Women vs. men	−0.22	0.235
	Female leader	Women vs. men	0.53	0.007
Lazy-hard-working	Women	Typical leader vs. male leader	0.56	***
		Typical leader vs. female leader	−0.42	0.001
		Male leader vs. female leader	−0.98	***
	Men	Typical leader vs. male leader	0.25	0.482
		Typical leader vs. female leader	0.07	1.000
		Male leader vs. female leader	−0.18	1.000
	Typical leader	Women vs. men	−0.04	0.833
	Male leader	Women vs. men	−0.35	0.095
	Female leader	Women vs. men	0.45	0.020

***p < 0.001; Bonferroni correction was used for interaction effects; positive differences indicate that the parameter on the left side of the comparison column is higher than the parameter on the right side of the comparison column and negative differences indicate that the parameter on the left side of the comparison column is lower than the parameter on the right side of the comparison column.

### 3.3. Evaluation of adjective pairs

#### 3.3.1. Interaction effects of stimulus and participants’ gender

Analyses showed a significant interaction between the within-subjects factor stimulus and the between-subjects factor participants’ gender for five evaluation scores. Evaluation scores of vacillating–persistent / inflexible–flexible, undiscriminating–selective / choosy–broad-minded, aggressive–peaceful / passive–forceful, and impractical–practical / opportunistic–idealistic revealed that women’s evaluation scores (i.e., how positively or negatively they rated the stimuli on the two corresponding adjective pairs) were equally high for typical and female leaders; however, women devalued male leaders as they indicated lower ratings of the evaluation scores for male leaders than men. Additionally, results on vacillating–persistent / inflexible–flexible and impractical–practical / opportunistic–idealistic showed that men evaluated typical and male leaders equally high; however, men devalued female leaders. No differences in men’s evaluations of the stimuli were found for undiscriminating–selective / choosy–broad-minded and aggressive–peaceful / passive–forceful. The analysis of the non-combined adjective pair lazy–hard-working showed that women evaluated female leaders the most hard-working, typical leaders the second most hard-working, and male leaders the laziest, while no differences in men’s evaluations were shown.

#### 3.3.2. Main effects of stimulus

Nine analyses on evaluation scores showed a significant effect for stimulus. Evaluation scores of frivolous–serious / grim–jolly, uncooperative–cooperative / conforming–independent, tactless–tactful / devious–frank, and deplorable–admirable / not likable–likable as well as the non-combined adjective pair stupid–intelligent indicated that typical and female leaders are evaluated equally and both are ranked higher in their evaluations than male leaders. Analyses on impulsive–self-controlled / inhibited–spontaneous, rash–cautious / timid–bold, and conceited–modest / unassured–self-confident revealed that typical leaders are evaluated the highest compared to both, male and female leaders; however, no differences between male and female leaders were found. Female leaders reached the highest evaluation for the evaluation score of extravagant–thrifty / stingy–generous, with typical leaders having the second highest evaluation, and male leaders the lowest evaluation.

#### 3.3.3. Non-significant effects

For the evaluation scores regarding gullible–skeptical / distrustful–trusting, lax–firm / severe–lenient, and agitated–calm / inactive–active no significant interaction or main effects for stimulus were found and none of the 17 analyses revealed significant main effects for participants’ gender. Table 2 shows the estimated marginal means and standard errors of evaluation scores for all groups and Table 3 shows information on test statistics for tests on evaluation scores. Mean differences and p values of pairwise comparisons are provided in Table 4.

## 4. Discussion

Previous research on gender stereotypes has widely used self-ratings on items created by researchers to analyze gender stereotypes, which do not allow for capturing stereotypes specific to individual participants. The present study aimed at gaining insights into the content of men and women’s actual thoughts and their evaluations of typical, male, and female leaders without using apparently gender-specific material. Social representations explicitly but subtly capture actual gender stereotypes and their evaluations, while the evaluative component of Peabody’s semantic differential implicitly measures participants’ evaluations. Results on social representations show that men and women ascribe typical leaders with many agentic and few communal characteristics in the central core. Core social representations for male leaders included mostly agentic characteristics, whereas female leaders’ central core consisted of agentic as well as communal characteristics. However, “empathic” (i.e., a communal characteristic) was the primary social representation of men to female leaders and men also reported “empathic” as a core social representation of typical leaders. Results on polarity indices (i.e., ratings of free associations) show that women evaluate female leaders more negatively than male and typical leaders. However, using the evaluative component of non-gendered adjective pairs show that typical and female leaders are often rated more positively than male leaders and that especially women devalued male leaders frequently. Accordingly, researchers and practitioners should consider whether explicit or implicit methods have been used to evaluate leaders and how prevailing gender stereotypes might influence these evaluations.

Results on typical and male leaders’ social representations reflect the assumptions of role congruity theory (Eagly and Karau, 2002). However, a slight change in the leadership stereotype toward having both masculine and feminine characteristics (Sczesny et al., 2004; Eagly and Sczesny, 2009; Koenig et al., 2011; Vinkenburg et al., 2011; Kark et al., 2012) could be argued. Gender stereotypes reflected thru social representations of male leaders did not change over time, contrary to expectations emerging from previous research on gender stereotypes of leaders (Rodler et al., 2001; Hartl et al., 2013). Core social representations of female leaders included agentic and communal characteristics; however, men’s primary social representation for female leaders was the communal characteristic “empathic.” This reduction of female leaders’ social representations to one preeminent characteristic could be interpreted as men’s prejudice and devaluation against female leaders (Eagly et al., 1992; Deal and Stevenson, 1998; Hoffmann and Musch, 2019) and also amplifies the double-bind expectations that female leaders face (Zheng et al., 2018). Women’s core social representations of typical leaders and female leaders are somewhat similar, with the associations “competent,” “assertive,” and “professional expertise” being represented in both central cores; however, also several representations being unique for either typical of female leaders were found. Men reported many social representations for typical leaders and only few for female leaders, with the social representations not matching, suggesting that men’s social representations of typical and female leaders differ substantially. This result is in line with previous research demonstrating that women see female and male leaders as equally competent for leadership positions (Schein, 2001; Boyce and Herd, 2003; Duehr and Bono, 2006; Berkery et al., 2013) and that men devalue female leaders (Eagly et al., 1992; Deal and Stevenson, 1998; Hoffmann and Musch, 2019).

To analyze social representations, free associations were used. Participants’ ratings of free associations were combined to polarity indices, indicating how negatively or positively the associations are rated and consequently also displaying whether the used stimuli are perceived as either positive or negative. In contrast to previous studies (Eagly et al., 1992; Deal and Stevenson, 1998; Hoffmann and Musch, 2019), women evaluated female leaders worse than typical and male leaders and men’s ratings did not differ between the stimuli. This indicates that women devalued female leaders. One explanation for this result might be that women’s associations for female leaders (e.g., have to prove themselves) considered female leaders’ tough work situations which were rated negatively; however, this explanation is contradicted by the fact that most associations from women to female leaders reflected characteristics rather than working situations.

Although most women evaluated agentic and communal characteristics associated with female leaders positively, some women made the same associations (e.g., empathic, assertive), but indicated negative ratings. For typical leaders, the same associations were stated, but neither men nor women evaluated them as negative. This disparity might reflect that agentic characteristics are seen as necessary for successful leadership and communal characteristics are a positive addition for typical leaders (Vial and Napier, 2018). However, for female leaders, the misfit of characteristics (Rudman et al., 2012; Williams and Tiedens, 2016) as well as their fit is punished (Eagly and Karau, 2002). An explanation could be women’s disapproval of masculine characteristics in leadership (Brenner et al., 1989; Paris et al., 2009; Stoker et al., 2012), which might be evaluated negatively, in particular, if they are displayed by female leaders. Women might also think that female leaders should not show too many “typical” feminine characteristics to be successful and thus rate communal characteristics for female leaders as negative.

Examining the evaluative component of adjective pairs demonstrates that typical leaders and female leaders are evaluated more positively compared to male leaders and that women frequently devalue male leaders. These results show a different pattern compared to results on ratings of associations; however, they still contradict the expectations from previous research that men devalue female leaders (Eagly et al., 1992; Deal and Stevenson, 1998; Hoffmann and Musch, 2019). When using non-gendered adjective pairs’ evaluative component female leaders were assessed more positively than when using evaluations of free associations. Thus, non-gendered adjective pairs might give a broader picture of leadership because they take the focus off stereotypes (i.e., implicit measurement of evaluations), as opposed to rating masculine and feminine characteristics of leaders (i.e., explicit measurement of evaluations). Directly asking about male and female leaders’ characteristics might retrieve participants’ representations on leaders’ gendered characteristics rather than on how male and female leaders are evaluated in general. Thus, stereotypes matching and stereotypes not matching to gender roles might be salient in this evaluation. When provided with non-gendered adjective pairs stereotypes might get in the background and the focus might shift to more general evaluations. Accordingly, using the evaluation component of adjective pairs might allow women to show their (subconscious) devaluation for male leaders as they are not directly asked to rate male leadership behavior.

Using social representations collected through free associations can capture stereotypes held by individual participants (*cf.* Eagly and Mladinic, 1989; Kite et al., 2008) and has several advantages over other methods (Wagner et al., 1999). They are less influenced by the material provided and thus participants reveal unstructured and latent views that are more unaffected by socially desired behavior (Kulich et al., 2005). Using obituaries (Rodler et al., 2001; Hartl et al., 2013) is also a subtle method to study what people think about male and female leaders. However, obituaries normally consider only positive aspects and are written for male and female leaders; thus, typical leaders or leaders in general cannot be examined. Using free associations also allows negative aspects of leaders to be surveyed; in addition, representations of typical leaders can be obtained. Peabody’s semantic differential with its evaluative component of adjective pairs is an implicit method to examine evaluations of stimuli. As participants are unaware of the underlying evaluations, socially desired answers are reduced and also more subconscious evaluations can be revealed. As the adjectives used are not characterized as mainly agentic or communal, evaluations are not only based on male and female stereotypes but also on other relevant characteristics and previous experiences. In addition, unlike other implicit measures, Peabody’s semantic differential is easier to apply as no response times are measured, the instructions are kept simple, and no experimental manipulation is required (*cf.* Greenwald and Banaji, 1995). The opposing patterns found for evaluations of associations and evaluations of adjective pairs reflect that direct questions generate mainly stereotyped answers, whereas asking indirectly might evoke more fundamental underlying responses.

### 4.1. Limitations and future research directions

The high dropout rate could not be examined regarding demographic data. Demographic data was collected at the end of the questionnaire and participants already dropped out before they answered these questions. In the questionnaire used for this study, participants were asked to produce their own statements and rate them afterwards as either negative, neutral, or positive, rather than presenting statements, which should be rated on Likert-type scales. Additionally, participants were asked to answer the same 32 adjective pairs for the three stimuli. Maybe participants dropped out because producing own statements was too exhausting and rating the same adjective pairs three times was too monotonous for them. It is noticeable that fewer men than women completed the questionnaire. One explanation for this imbalance could be that fewer men than women started the questionnaire because they were less often addressed or they were less interested in the research topic than women were. Another explanation could be that men had a higher dropout rate than women. Maybe men disliked specific parts of the questionnaire or found answering the questions especially effortful and thus discontinued answering.

In this paper parametric tests were conducted to examine underlying interaction effects; however, several assumptions for parametric testing were violated. Although analyses of variance are robust to violations of assumptions, these violations could compromise the found results. Thus, future research should use nonparametric alternatives to analyze mixed analyses of variance.

Further limitations concern the lack of randomization of the adjective pairs and the repeated tasks in the questionnaire. Although the stimuli were presented in a random order generated by the system in the first phase of the questionnaire, this order was maintained in the second phase. In addition, adjective pairs were presented in the same order and format (i.e., negatively rated adjectives on the left side and positively rated adjectives on the right side) for all stimuli. This lack of randomization could lead to a common method bias, especially since the tasks of producing associations and rating adjective pairs were repeated three times in a similar manner. Participants may have been trying to expedite their response time and therefore refrained from reading the adjective pairs carefully or thinking about their answers. However, each stimulus was answered as the first stimulus by one-third of the participants, which addresses at least some concerns.

As the spontaneously produced associations included synonyms, associations with the same meaning were allotted to one association (i.e., aligning the synonyms); however, it is subjective whether words are synonyms or have a slightly different meaning. Thus, the rater’s interpretation regarding synonyms influences the associations’ frequency, and consequently which associations are included in the central core. Further, thresholds which divided the central core and peripheral elements were set by the authors after careful considerations; however, the used thresholds affect the obtained results.

Using social representations collected with free associations revealed the predominant gender stereotypes of typical, male, and female leaders and showed that women devalue female leaders. By contrast, using ratings of non-gendered adjective pairs showed that typical and female leaders are evaluated more positively than male leaders and that women often devalue male leaders. Free associations are collated with explicit questions, whereas non-gendered adjective pairs contain an evaluative component, which is an implicit measure. When studying gender stereotypes in leadership, future research should distinguish between explicit and implicit measures to better scrutinize underlying effects.

Previous studies found that asking people who had prior work experiences with female leaders led to less stereotypical characterizations of leaders than asking student samples who did not have prior work experiences with female leaders (Eagly and Johnson, 1990; Koenig et al., 2011; Berkery et al., 2013). Thus, in the present study, only participants with work experience took part. A promising addition to the present study would be to compare associations and evaluations of participants who had prior experiences with female leaders to answers from participants without such experiences. Moreover, comparing associations of women and men in leadership positions could provide more specific information.

It is important to point out that only associations and evaluations based on stimuli were examined and that those do not reflect leaders’ behavior. Future studies should consider leaders’ evaluations on their own agentic and communal characteristics as well as their actual behavior. Moreover, comparing leaders’ self-descriptions to descriptions of their employees as well as general descriptions of leaders would be promising for future research.

### 4.2. Practice implications

Findings from the current study have implications for human resource policymakers and employment advisers. Human resource managers are responsible for fair procedures in recruiting, personnel development, performance measurement, and career advancements. For maintaining fair procedures regarding gender, it is important that decision makers are aware their evaluation methods regarding male and female leaders might be influenced by gender stereotypes.

Results show that gender stereotypes for male and female leaders converge for both having agentic and communal characteristics as core social representations; however, gender stereotypes still prevail. Especially, men’s social representations of female leaders being almost exclusively “empathic” could be problematic for women’s careers as men are the main gatekeepers for women’s advancements. To install fair procedures, which are not influenced by prevailing gender stereotypes, human resource policymakers should draw organizations’ attention to the misfit of characteristics. As a first step, introducing standardized procedures in human resource processes and equality plans, in which women have similar career opportunities to men, could further fair procedures.

Another finding relevant for human resource managers’ evaluations is that social representations of typical leaders not only include agentic characteristics but also communal characteristics. Thus, selecting leaders who show both agentic and communal characteristics might be crucial in future hiring processes. In addition, male and female leaders who show mainly stereotypical characteristics could be coached to acquire missing non-stereotypical characteristics, which are nevertheless essential for leadership.

The finding that typical and female leaders differ in some but also share some core social representations is also important when coaching women who want to advance in their workplace. Employment advisers should draw women’s attention to the differences and similarities in social representations of typical, male, and female leaders. However, coaching women to show more agentic and less communal characteristics could backfire, as women showing agentic characteristics are frequently devalued (Rudman et al., 2012; Williams and Tiedens, 2016; Ferguson, 2018). Women could be advised to insist on standardized evaluations, in which their performance and the performance of their male counterparts can be compared directly. These direct comparisons could counteract unfair decisions.

## 5. Conclusion

The present study examines gender stereotypes regarding leaders through social representations as well as through evaluations of free associations and non-gendered adjective pairs. With these approaches it was demonstrated that social representations of typical and female leaders consist of both agentic and communal characteristics; however, men’s social representations of female leaders refer almost exclusively to the communal characteristic “empathic.” This result could explain why female leaders face difficulties in moving up organizational ranks and being discriminated against in varying organizational decisions. They are not ascribed leadership qualities but are mostly seen as “empathic” by men who are often in higher positions (Grant Thornton, 2020; Mercer, 2020) and thus responsible for decisions in these areas. Comparing findings of the explicit ratings of free associations and of the implicit evaluative component of non-gendered adjectives revealed a contradicting pattern. Women’s free associations elicit negatively rated representations of female leaders, whereas women’s ratings on non-gendered adjective pairs reveal women’s negative evaluations of male leaders. Leadership research should further scrutinize how explicit and implicit evaluation methods of leaders are affected by gender stereotypes. From this, methods could be developed which are less influenced by gender stereotypes.

## Data availability statement

The datasets presented in this study can be found in online repositories. The data and supplementary material can be found at: https://data.mendeley.com/datasets/2677g3pjmf.

## Ethics statement

Ethical review and approval were not required for the study on human participants in accordance with the local legislation and institutional requirements. Written informed consent for participation was not required for this study in accordance with the national legislation and the institutional requirements.

## Author contributions

All authors listed have made a substantial, direct, and intellectual contribution to the work and approved it for publication.

## Conflict of interest

The authors declare that the research was conducted in the absence of any commercial or financial relationships that could be construed as a potential conflict of interest.

## Publisher’s note

All claims expressed in this article are solely those of the authors and do not necessarily represent those of their affiliated organizations, or those of the publisher, the editors and the reviewers. Any product that may be evaluated in this article, or claim that may be made by its manufacturer, is not guaranteed or endorsed by the publisher.
